# Etymologia: Zika Virus

**DOI:** 10.3201/eid2006.ET2006

**Published:** 2014-06

**Keywords:** etymologia, Zika virus, viruses, Uganda, flavivirus, mosquitoborne, macaque

## Zika [zēkʹə] Virus

Zika virus is a mosquito-borne positive-sense, single-stranded RNA virus in the family *Flaviviridae*, genus *Flavivirus* that causes a mild, acute febrile illness similar to dengue. In 1947, scientists researching yellow fever placed a rhesus macaque in a cage in the Zika Forest (*zika* meaning “overgrown” in the Luganda language), near the East African Virus Research Institute in Entebbe, Uganda. A fever developed in the monkey, and researchers isolated from its serum a transmissible agent that was first described as Zika virus in 1952. It was subsequently isolated from a human in Nigeria in 1954. From its discovery until 2007, confirmed cases of Zika virus infection from Africa and Southeast Asia were rare. In 2007, however, a major epidemic occurred in Yap Island, Micronesia. More recently, epidemics have occurred in Polynesia, Easter Island, the Cook Islands and New Caledonia.
